# Phylogeny and expression analysis of *C-reactive protein* (*CRP*) and *serum amyloid-P* (*SAP*) like genes reveal two distinct groups in fish

**DOI:** 10.1016/j.fsi.2017.03.037

**Published:** 2017-06

**Authors:** P.T. Lee, S. Bird, J. Zou, S.A.M. Martin

**Affiliations:** a Scottish Fish Immunology Research Centre, Institute of Biological and Environmental Sciences, University of Aberdeen, Aberdeen AB24 2TZ, UK; b Science & Engineering, University of Waikato, Private Bag 3105, Hamilton 3240, New Zealand

**Keywords:** Acute phase protein, C-reactive protein, Serum amyloid-P, Atlantic salmon, Innate immunity, Gene expression

## Abstract

The acute phase response (APR) is an early innate immune function that is initiated by inflammatory signals, leading to the release of acute phase proteins to the bloodstream to re-establish homeostasis following microbial infection. In this study we analysed the Atlantic salmon (*Salmo salar*) whole-genome database and identified five *C-reactive protein (CRP)*/*serum amyloid P component (SAP)* like molecules namely *CRP/SAP-1a*, *CRP/SAP-1b*, *CRP/SAP-1c*, *CRP/SAP-2* and *CRP/SAP-3*. These CRP/SAP genes formed two distinct sub-families, a universal group (group I) present in all vertebrates and a fish/amphibian specific group (group II). Salmon *CRP/SAP-1a*, *CRP/SAP-1b* and *CRP/SAP-1c* and *CRP/SAP-2* belong to the group I family whilst salmon *CRP/SAP-3* is a member of group II. Gene expression analysis showed that the salmon *CRP/SAP-1a* as well as *serum amyloid A-5* (*SAA-5*), one of the major acute phase proteins, were significantly up-regulated by recombinant cytokines (rIL-1β and rIFNγ) in primary head kidney cells whilst the other four *CRP/SAPs* remained refractory. Furthermore, *SAA-5* was produced as the main acute phase protein (APP) in Atlantic salmon challenged with *Aeromonas salmonicida* (aroA(-) strain) whilst salmon *CRP/SAPs* remained unaltered. Overall, these data illustrate the potential different functions of expanded salmon *CRP/SAPs* to their mammalian homologues.

## Highlights

• Five pentraxin-like molecules are identified in the Atlantic salmon genome.
• The phylogenetic suggests two main groups of CRP/SAP genes.
• The expression of these genes has been studied *in vitro* following recombinant cytokine stimulation in head kidney cells.
• CRP/SAP gene expression studied in liver following bacterial infection.
• Possible models of metazoan and teleost fish SAP/CRP evolution are discussed.

## 1 Introduction

The acute phase response (APR), a core part of the innate immune system, comprises a series of physiological and biochemical reactions including change of body temperature, vascular permeability, electrolyte levels, bone marrow-derived cells and acute phase proteins (APPs) production, which aims to re-establish homeostasis during an inflammatory response [5], [15]. The proinflammatory cytokines including interleukin-1 (IL-1), IL-6 and tumour necrosis factor-α (TNF-α), are known to affect numerous signalling pathways throughout the body and serve as major factors for the initiation of the APR [10]. Key APPs in vertebrates, including α2-macroglobulin, serum amyloid A (SAA), serum amyloid P (SAP), C-reactive protein (CRP), mannose binding lectin (MBL) and complement components, are thought to be involved in several processes to neutralise microbial infection [15], [29].

The SAA genes have been identified in the human and mouse genome, with 4 and 5 copies sequenced in each species [15]. The human SAA1 and SAA2 and murine SAA1, SAA2 and SAA3 are highly inducible in hepatocytes during inflammation and are categorised as acute SAAs (A-SAAs). The synthesis of A-SAAs can be accelerated by proinflammatory cytokines that are released at the site of pathology, resulting in a number of innate immune processes such as chemotaxis of polymorphonuclear cells, monocytes, and T cells [5], [26], [29], immune cells priming [2], opsonisation of Gram-negative bacteria [39] and cytokine production [32]. Human SAA4 and mouse SAA5 are constitutively expressed and their functions still remain unclear. Human SAA3 and murine SAA4 are pseudogenes [42]. A single copy of *SAA* gene has been characterised in several teleost species, such as Atlantic salmon (*Salmo salar*) [17], rainbow trout (*Oncorhynchus mykiss*) [36], [48], Arctic char (*Salvelinus alpinus*) [15] and goldfish (*Carassius auratus*) [19] and its expression has been consistently shown to be increased in response to bacterial pathogens or bacterial related pathogen associated molecular patterns (PAMPs), confirming that fish SAA is a major APP.

Pentraxins including CRP and SAP are a superfamily of proteins that are characterised by a 200 amino acid pentraxin domain at the carboxyl-terminus. They are well conserved in arthropods, fish, amphibians, and mammals [9], [40]. Although sharing strong sequence homology, CRP and SAP exhibit different ligand binding specificities. For example, human CRP is known to specifically bind to the phosphocholine residues of the C-polysaccharide of the cell wall of *Streptococcus pneumonia* [45] whilst SAP (named for its presence in amyloid deposits) binds to phosphoethanolamine (PE) [38] and pyruvate acetal of agarose [13] but not phosphorylcholine. The basal protein levels of CRP and SAP and alteration of serum levels during inflammation may vary significantly in different mammalian species. Human CRP was massively increased following infection with no changes of SAP observed. In contrast, murine SAP is the major APP whilst CRP is merely a trace protein [35]. Pentraxin-like proteins are ubiquitously present in all fish species examined to date and have been named according to the binding specificities comparable to the known mammalian CRP/SAP ligands. However, the phylogeny and potential roles as APP are poorly investigated.

In this paper, we identified five pentraxin-like molecules in the Atlantic salmon genome. Based on the phylogenetic analyses, a nomenclature was proposed for fish molecules. We also analysed the expression profiles of salmon *CRP/SAP*-like molecules and *SAA-5* in primary head kidney cells treated with cytokines and in liver of bacterial infected fish.

## 2 Materials and methods

### 2.1 Sequence retrieval and analyses

The Atlantic salmon whole-genome [24] was BLAST screened using the salmon pentraxin-like molecules (Accession nos. NP_001134140 and NP_001117143) as queries. Amino acid sequence alignment of selected vertebrate CRP and SAP homologues was performed using ClustalW program (version 2.0) [12] (http://www.ebi.ac.uk/Tools/msa/clustalw2/). and phylogenetic tree construction, using the Neighbour-joining algorithm, was carried out with the MEGA6 program [44] (http://www.megasoftware.net/). The Poisson model was applied for amino acid substitution and pairwise deletion for gaps treatment. The tree was supported by 10,000 bootstrap repetitions. Deduced protein domain architectures was analysed using the Simple Modular Architecture Research Tool (SMART) [23] (http://smart.embl-heidelberg.de/) and the presence of a signal peptide was further confirmed using the SignalP program (version 4.1) (http://www.cbs.dtu.dk/services/SignalP/). Information on the organisation of CRP/SAP genes was obtained from the Ensembl (www.ensembl.org/), UniPort, Genomicus database (version 86.01) [25] and NCBI (http://ncbi.nlm.nih.gov) databases.

### 2.2 Experimental fish

Tissue samples including gills, muscle, liver, head kidney and spleen from 3 healthy Atlantic salmon parr (21.7 ± 1.8 g) were obtained as described previously [22] and used to examine tissue distribution of gene expression. For bacterial infection, juvenile salmon parr (average weight 67.4 ± 2.2 g) (mixed sex) were anaesthetised with benzocaine (Sigma-Aldrich, 20 mg l^−1^) and injected intraperitoneally (i.p.) with 100 μl of a genetically attenuated (aro A-) strain of *Aeromonas salmonicida* (Brivax II [30], (10^9^ CFU ml^−1^) or 100 μl of PBS as control [31] (n = 8 for both infected and control samples). For the response to infection fish were killed 24 h post challenge and liver samples were collected, stored in RNAlater (Ambion) at 4 °C for 24 h and then placed at −20 °C until RNA extraction. All procedures were conducted in accordance with the UK Home Office ‘Animals and Scientific Procedures Act 1986’.

### 2.3 *In vitro* expression study

Preparation of head kidney cells and cytokine treatment were performed as described previously [22]. The isolated cells were treated with fresh complete medium (Leibovitz's L-15 medium (Gibco) containing 10% (v/v) foetal calf serum (FCS, Sigma-Aldrich), penicillin (100 U/ml)/streptomycin (100 μg/ml) (P/S) mix (Gibco)) alone or medium containing recombinant IFNγ (20 ng/ml) [49] or IL-1β (20 ng/ml) [50]. The cells were maintained in culture for 4 h, 8 h, 24 h and 48 h and harvested for RNA extraction by lysis with TRI-Reagent (Sigma-Aldrich).

### 2.4 RNA extraction, cDNA synthesis and real time PCR (RT-PCR)

Total RNA extraction and cDNA synthesis were performed as described previously [22]. Briefly, total RNA was extracted from the tissue samples and primary head kidney leukocytes using TRI-Reagent. DNase I (Ambion) treatment was carried out according to the manufacturer's instructions to remove genomic DNA contamination. Reverse transcription was conducted using a RevertAid™ First Strand cDNA Synthesis Kit (Thermo Scientific) and cDNAs were diluted with 400 μl TE buffer (pH 8.0) prior to be analysed by real-time PCR using primers listed in Table 1.

**Table 1:** Gene primer information.

Gene name	Forward primer (5′-3′)	Reverse primer (5′-3′)	Accession number
EF-1α	CAAGGATATCCGTCGTGGCA	ACAGCGAAACGACCAAGAG	AF321836
β-actin	AAGATGAAATCGCCGCAC	ATGGAGGGGAAGACAGCC	AF012125
Hprt1	CCGCCTCAAGAGCTACTGTAAT	GTCTGGAACCTCAAACCCTATG	BT043501
SAA-5	GGTGAAGCTGCTCGAGGTGC	CCATCTCCCGGCCATTACTGAT	NM_001146565
CRP/SAP-1a	GTTATGGTGAACATCAAGATCTC	CATGAGACTGGGTTGCCAGA	NM_001123671.1
CRP/SAP-1b	GTTATGGTGAACCTCAAGATCTCT	GGAATCAGAGGGGGTTGCTAG	NM_001140668.1
CRP/SAP-1c	CTGGGGATTGCCAGACAAAA	TATGCATCTTGGGCCTGACC	XM_014171429.1
CRP/SAP-2	ACTGCTGGGAGTGTGCTGAA	CGAGAACCACATGCCCATCAA	XM_014199767.1
CRP/SAP-3	CCTCAGCCAATAAAGTTGGCC	AGGCGAACAGGATGATCTGC	XM_014158882.1
IL-1β	CCGTCCCCATTGAGACTAAAG	TGTCGCTCTGCTGGCTGA	AY617117
IL-8	GAATGTCAGCCAGCCTTGTC	TCCAGACAAATCTCCTGACCG	NM_001140710
TNF-α1/2	ATGGAAGACTGGCAACGATG	TCACCCTCTAAATGGATGGC	AY929385+AY929386

Primers used in real-time PCR analysis were designed as follow: For salmon *SAA-5*, primers were designed to cross the intron-exon junctions. The salmon *CRP/SAP* cDNA sequences were first aligned and examined manually to ensure the specificity of all the primer sets and cross the intron-exon junctions where possible (Supplementary Fig. 1). The amplification of each *CRP/SAP* gene was confirmed by examining the melting curve and product size.

For analysis of gene expression, three housekeeping genes including *elongation factor-1α* (*EF-1α*), *β-actin* and *hypoxanthine phosphoribosyl transferase 1* (*Hprt1*), were used for normalisation. The relative expression profile of salmon *CRP/SAP* homologues and *SAA-5* from five different tissues sampled from Atlantic salmon parr (n = 3) was normalised to the geometric mean of the Ct values of three housekeeping genes (*EF-1α*, *β-actin* and *Hprt1*) and expressed as a fold-change relative to gill. For *in vitro* experiments, the expression level of *CRP/SAPs* and *SAA-5* in cells treated with medium (n = 3) or medium with IL-1β (n = 3) or IFNγ (n = 3) was measured. The relative fold changes of target genes were obtained by first normalising to the geometric mean of the Ct values of the three housekeeping genes and the data then presented as relative expression of the treated groups to the corresponding control groups. To examine regulation during disease challenge, the expression level of *CRP/SAPs* and *SAA-5* in *A. salmonicida* challenged fish (n = 8) was normalised to the three housekeeping genes (*EF-1α*, *β-actin* and *Hprt1*) and expressed as a fold-change relative to the uninfected control fish (n = 8).

### 2.5 Statistical analysis

Gene expression results were analysed by *t*-test. Primers efficiency was set as 1. *P* < 0.05 was considered significant (expression data is presented as mean ± standard error).

## 3 Results

### 3.1 Sequence analysis of salmon CRP/SAP-like molecules

**Fig. 1:**
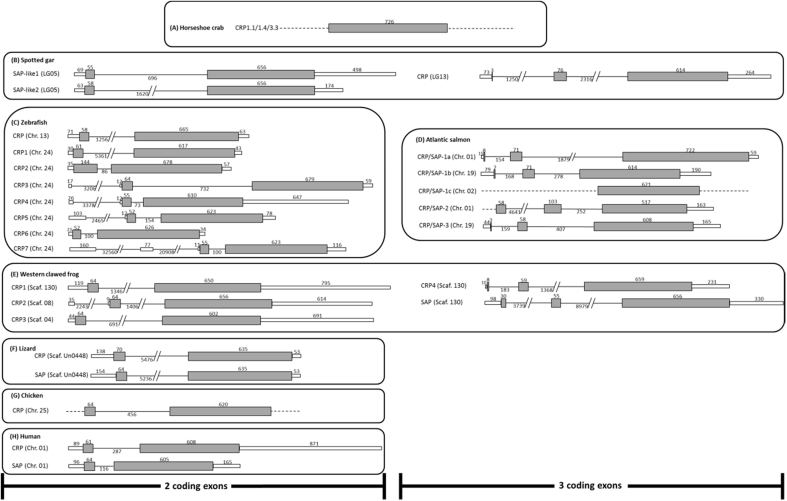
Genomic structure of (A) horseshoe crab, (B) spotted gar, (C) zebrafish, (D) Atlantic salmon, (E) western clawed frog, (F) lizard, (G) chicken, and (H) human CRP/SAP genes. The white and the grey bars represent untranslated region (UTR) and exon respectively. Introns are indicated by black lines. The sizes (bp) of UTRs, exons and introns are numbered and drawn in scale except for some introns presented with//. Sequences with unknown size were shown by dash lines.

Two pentraxin-like genes identified on chromosome 1 were named *CRP/SAP-1a* and *CRP/SAP-2*, respectively (see below for nomenclature). The salmon *CRP/SAP-1a* (Ssa01 LOC100136583 (53389000–53391897), NM_001123671.1) is comprised of 3 exons and 2 introns with a putative ORF of 801bp and a partial 15bp 5′ untranslated region (UTR) and a 59bp 3′ UTR (Fig. 1 and Supplementary Fig. 1). The deduced salmon *CRP/SAP-2* (Ssa01 LOC106604759 (49435076–49436975), XM_014199767.1) also consists of 3 exons and 2 introns, with a putative ORF of 678bp and an additional 163bp 3′ UTR (Fig. 1 and Supplementary Fig. 1). On chromosome 19, two pentraxin-like molecules were identified and termed *CRP/SAP-1b* (Ssa19 Loc100195639 (57275409–57297491, complement), NP_001134140), and *CRP/SAP-3* (Ssa19 LOC106579201 (54094913–54097615, complement), XM_014158882.1), respectively. Both genes have 3 exons and 2 introns with a putative ORF of 693 bp and 669 bp, respectively (Fig. 1 and Supplementary Fig. 1). On chromosome 2, a single exon gene was predicted (Ssa02 LOC106585334 (40009653–40010851, complement), XM_014171429.1) and termed salmon *CRP/SAP-1c* (Fig. 1 and Supplementary Fig. 1).

**Fig. 2:**
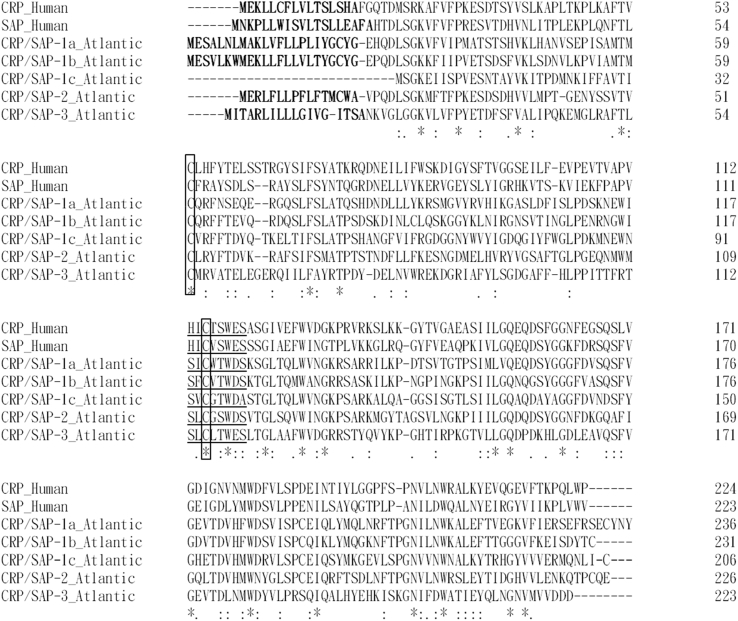
Alignment of deduced amino acid sequences of salmon CRP/SAP-1a, CRP/SAP-1b, CRP/SAP-1c, CRP/SAP-2 and CRP/SAP-3 and human homologues. The predicted signal peptide is in bold text. The typical pentraxin domains (H/SxCxS/TWxS, x represents any amino acids) are underlined and two conserved cysteines are boxed.

Several sequence features including a signal peptide (except for CRP/SAP-1c), a classical pentraxin signature (H/SxCxS/TWxS, x represents any amino acids) and two conserved cysteines are found in the salmon CRP/SAPs (Fig. 2), suggesting that they are typical CRP/SAP molecules. The genomic structure of three coding exons is conserved for most salmon CRP/SAP-like genes (except for *CRP/SAP-1c*), spotted gar *CRP*, and frog *SAP* and *CRP4* genes. In contrast, the *CRP/SAP* genes of human, chicken, lizard, and zebrafish, spotted gar *SAP-like1* and *SAP-like2* and frog *CRP1*, *CRP2* and *CRP3* consisted of two coding exons (Fig. 1).

### 3.2 Phylogenetic analysis of salmon CRP/SAPs

**Fig. 3:**
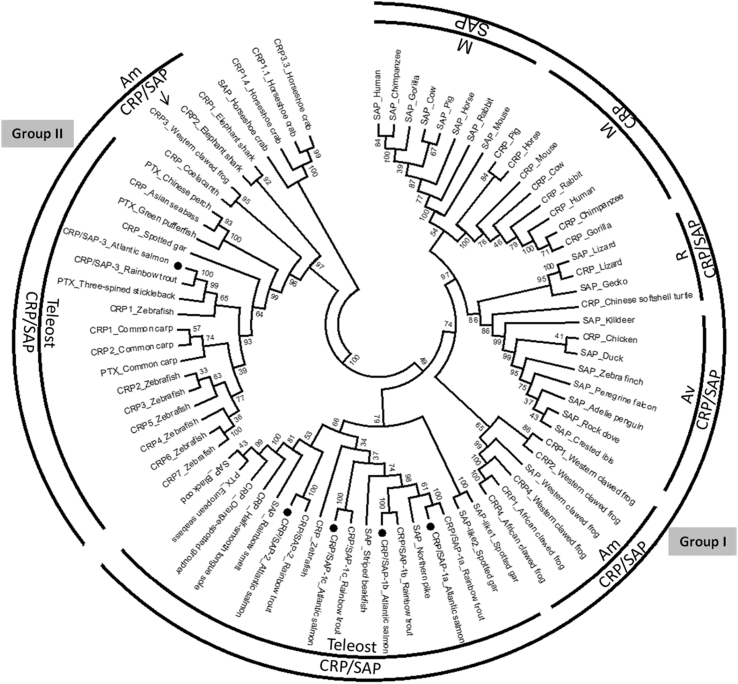
A rooted phylogenetic tree of Atlantic salmon C-reactive protein (CRP)/serum amyloid P (SAP) homologues with selected CRP/SAP and short pentraxin (PTX) molecules. Amino acid sequences were aligned using the ClustalW programme and the tree was built using the Neighbour-joining method in the MEGA 6 program. The tree was supported by 10,000 bootstrap replications using Poisson correction and pairwise deletion of gaps. M: mammalian; R: reptile; Av: avian; Am: amphibian. Accession numbers of the sequences used are as follows: for human-SAP: NP_001630, CRP: AAA52075; for chimpanzee-SAP: XP_513916, CRP: XP_001170732; for gorilla-SAP: XP_004027117, CRP: XP_004027118; for rabbit-SAP: XP_002715334, CRP: AAA75404; for murine-SAP: NP_035448, CRP: CAA31928; for cow-SAP: NP_001029638, CRP: NP_001137569; for pig-SAP: NP_999052, CRP: NP_999009; for horse-SAP: ENSECAT00000007982, CRP: ENSECAT00000026823; for chicken-CRP: NP_001034653; for killdeer-SAP: XP_009879105; for zebra finch-SAP: XP_002198443; for rock dove-SAP: XP_005509056; for peregrine falcon-SAP: XP_005244420; for adélie penguin-SAP: KFW65736; for crested ibis-SAP: XP_009463234; for duck-SAP: ENSAPLT00000001066; for Chinese softshell turtle-CRP: XP_006115298; for gecko-SAP: XP_015280436; for lizard-CRP: XP_008120254, SAP: XP_003228449; for western clawed frog-SAP: NP_001008175, CRP1: XP_002934127, CRP2: OCA26837, CRP3: XM_002935612; CRP4: ENSXETT00000008791; for African clawed frog-CRP1: NP_001165686, CRP4: NP_001085945; for coelacanth-CRP: XP_006011428; for rainbow trout-CRP/SAP-1a: NP_001118193, CRP/SAP-1b: CDQ63622, CRP/SAP-1c: CDQ61378, CRP/SAP-2: CDQ61095, CRP/SAP-3: CDQ58740; for northern pike-SAP: ACO14371; for striped beakfish-SAP: BAM36369; for rainbow smelt-SAP: ACO09135; for half-smooth tongue sole-CRP: JX914666; for orange-spotted grouper-CRP: ADC92292; for European seabass-pentraxin: C6ETM3; for black cod-SAP: ACQ58218; for Chinese perch-pentraxin: D3TJK9; for Asian seabass-CRP: HQ652974; for green pufferfish-PTX: Q4SAT8; for three-spined stickleback-pentraxin: B5AQ36; for zebrafish-CRP: ENSDART00000131351, CRP1: ENSDART00000105662, CRP2: ENSDART00000056381, CRP3: ENSDART00000056382, CRP4: NM_001040297 CRP5: ENSDART00000147783, CRP6: ENSDART00000105666, CRP7: ENSDART00000109975; for common carp-pentraxin: Q90YD1, CRP1: JQ010977, CRP2: JQ010978; for spotted gar-CRP: XP_015215488, SAP-like1: XP_006631034, SAP-like2:XP_006631035; for elephant shark-CRP1: XP_007883122, CRP2: XP_007883123; for Atlantic horseshoe crab-CRP1.1: AAA28270, CRP1.4: M14024, CRP3.3: P06207; SAP: AY066022.

To examine the phylogenetic relationship of salmon CRP/SAP molecules, a phylogenetic tree was built using the Neighbour-joining method based on the multiple alignment of amino acid sequences and rooted with the Atlantic horseshoe crab CRP protein (Fig. 3). In the tree, fish and frog CRP/SAP sequences are well separated into two distinct clades. The first clade (termed Group I) contains CRP/SAP molecules from mammals, reptiles, birds, amphibians and several piscine species, whilst the second clade (termed Group II) comprises a number of fish and amphibian CRP/SAP molecules (Fig. 3). Three of salmon CRP/SAP molecules (CRP/SAP-1a, CRP/SAP-1b, and CRP/SAP-1c) belong to the fish group I CRP/SAP family whilst salmon CRP/SAP-3 is a member of fish group II CRP/SAPs. Amphibians also have group I (CRP1, CRP2, CRP4 and SAP) and II (CRP3) CRP/SAPs.

### 3.3 Comparative genome synteny of salmon *CRP/SAPs*

**Fig. 4:**
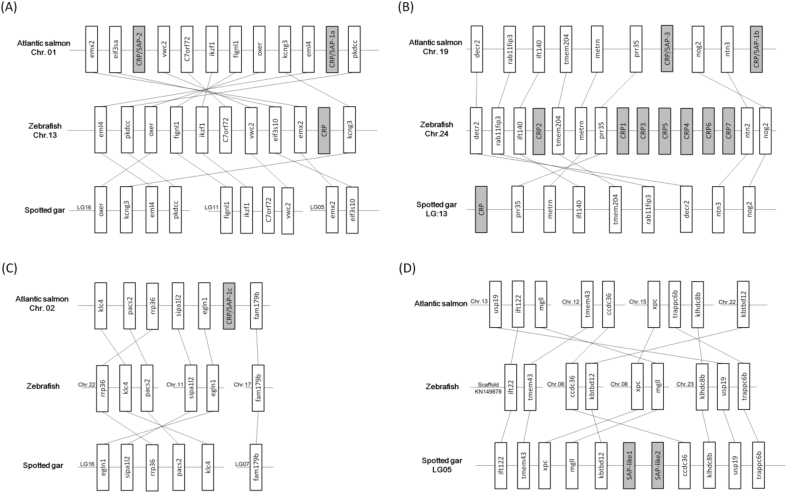
Comparison of the genome syntenic regions of CRP/SAP homologues from Atlantic salmon, spotted gar, zebrafish and humans. Gene order for spotted gar, zebrafish and human genes were obtained from the Genomicus database (version 84.01) (http://www.genomicus.biologie.ens.fr/genomicus-84.01/cgi-bin/search.pl). Gene order for Atlantic salmon was obtained from NCBI (https://www.ncbi.nlm.nih.gov/genome/?term=atlantic+salmon). Gene distance is not drawn to scale. CRP, C-reactive protein; SAP, serum amyloid-P; VWC2, von Willebrand factor C domain-containing protein 2; C7orf72, uncharacterized protein C7orf72 homolog; IKZF1, DNA-binding protein Ikaros; FIGNL1, fidgetin-like protein 1; OXER, oxoeicosanoid receptor; KCNG3, potassium voltage-gated channel subfamily G member 3; EML4, echinoderm microtubule-associated protein-like 4; PKDCC, extracellular tyrosine-protein kinase PKDCC; EMX2, homeobox protein EMX2; EIF3A, eukaryotic translation initiation factor 3 subunit A; NTN3, Netrin 3; NOG2, noggin 2; PPR35, proline rich 35; METRN, meteorin; TMEM204, transmembrane protein 204; IFT140, intraflagellar transport protein 140 homolog; RAB11FIP3, RAB11 family interacting protein 3; DECR2, 2,4-dienoyl-CoA reductase 2, peroxisomal; KLC4, kinesin light chain 4; PACS2, phosphofurin acidic cluster sorting protein 2; RRP36, ribosomal RNA processing protein 36 homolog; SIPA1L2, signal-induced proliferation-associated 1-like protein 2; EGLN1, egl nine homolog 3-like; FAM179B, protein FAM179B; IFT22, intraflagellar transport 122; TMEM43, transmembrane protein 43; XPC, DNA repair protein complementing XP-C cells, mgll, monoglyceride lipase-like; KBTBD12, kelch repeat and BTB domain-containing protein 12; ccdc36, coiled-coil domain-containing protein 36; KLHDC8B, kelch domain-containing protein 8B; USP19, ubiquitin carboxyl-terminal hydrolase 19; trappc6b, trafficking protein particle complex subunit 6B.

The salmon *CRP/SAP* genes are located on three chromosomes, Chr. 01, 02 and 19 (Fig. 4). The genes adjacent to salmon *CRP/SAP-1a* and *CRP/SAP-2* can also be found in the homologous locus in the zebrafish genome (Chr.13). Genes including *von Willebrand factor C domain-containing protein 2* (*VWC2*), *uncharacterized protein C7orf72 homologues* (*C7orf72*), *DNA-binding protein Ikaros* (*IKZF1*) and *fidgetin-like protein 1* (*FIGNL1*) were found on LG11 in spotted gar, and other neighbouring genes such as *oxoeicosanoid receptor* (*OXER*), *potassium voltage-gated channel subfamily G member 3* (*KCNG3*), *echinoderm microtubule-associated protein-like 4* (*EML4*), and *extracellular tyrosine-protein kinase PKDCC* (*PKDCC*) were found located on another locus for spotted gar (LG16). No homologous genes to salmon *CRP/SAP* were found in the genome segments of spotted gar (LG05, 11 and 16) (Fig. 4A). However, the genome segment containing salmon *CRP/SAP-1b* and *CRP/SAP-3* (Chr. 19) exhibits conserved synteny to the corresponding genome segments from zebrafish (Chr. 24, containing 7 copies) and spotted gar (LG13, containing a single copy) (Fig. 4B). The homologous genome loci of zebrafish and spotted gar lack any *CRP/SAP* genes compared with that of the salmon *CRP/SAP-1c* gene locus (Chr. 02) (Fig. 4C). Similarly, the synteny of genome segment containing gar *SAP-like1* and *SAP-like2* genes (LG05) is less conserved in zebrafish and Atlantic salmon.

### 3.4 Tissue distribution of salmon CRP/SAPs and SAA in Atlantic salmon

**Fig. 5:**
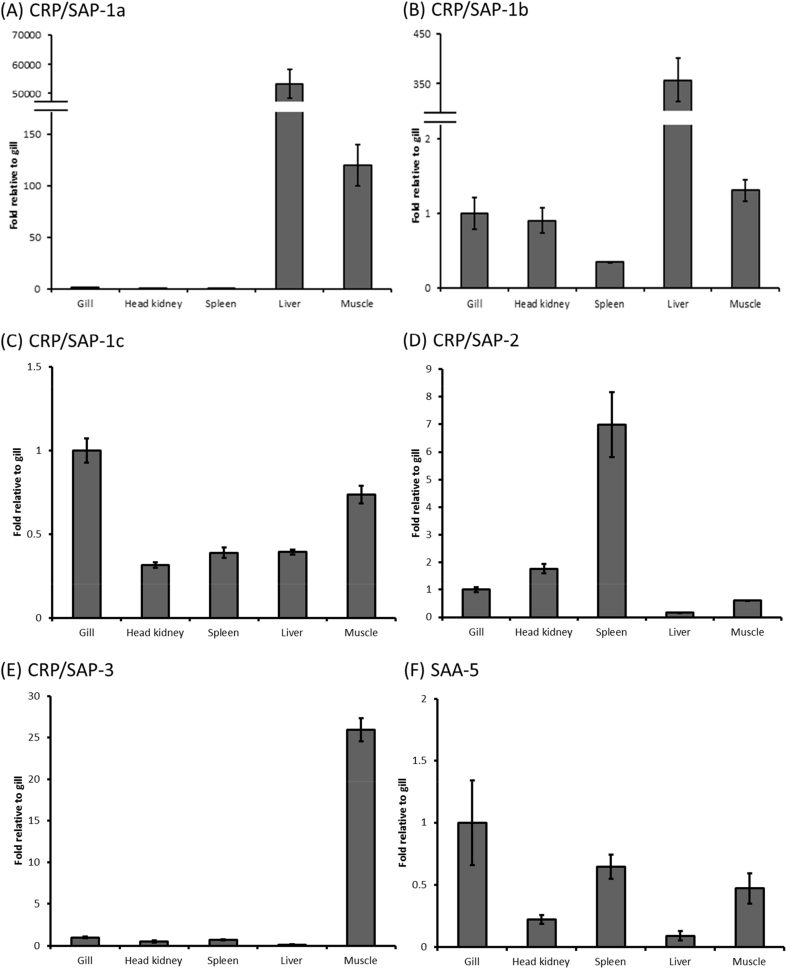
Relative gene expression of CRP/SAP homologues and *SAA-5* in healthy tissues of Atlantic salmon parr. The relative expression levels of CRP/SAP homologues and SAA-5 in tissue samples were normalised to the expression levels of three housekeeping genes (*EF-1α*, *β-actin* and *Hprt1*) and expressed as a fold-change relative to gill for *CRP/SAP-1a* (Ct = 28.7), *CRP/SAP-1b* (Ct = 24.4), *CRP/SAP-1c* (Ct = 30.7), *CRP/SAP-2* (Ct = 22.9), *CRP/SAP-3* (Ct = 29.1) and *SAA-5* (Ct = 21.9). Results are means ± SE of 3 individual fish.

Tissue samples including gills, spleen, liver, muscle, and head kidney from healthy Atlantic salmon parr were used to detect the basal expression of *CRP/SAP* and *SAA* genes. The expression of salmon *CRP/SAP-1a* and *CRP/SAP-1b* was highly expressed in liver (Fig. 5A and Fig. 5B). In contrast, *CRP/SAP-2* and *CRP/SAP-3* was expressed the highest in spleen and muscle respectively but relatively lowly expressed in liver tissue (Fig. 5D and E). For the salmon *CRP/SAP-1c* and *SAA-5* genes, they were highly expressed in gills, followed by spleen and muscle tissue (Fig. 5C and F).

### 3.5 Expression of *CRP/SAPs* and *SAA-5* in vivo and vitro

**Fig. 6:**
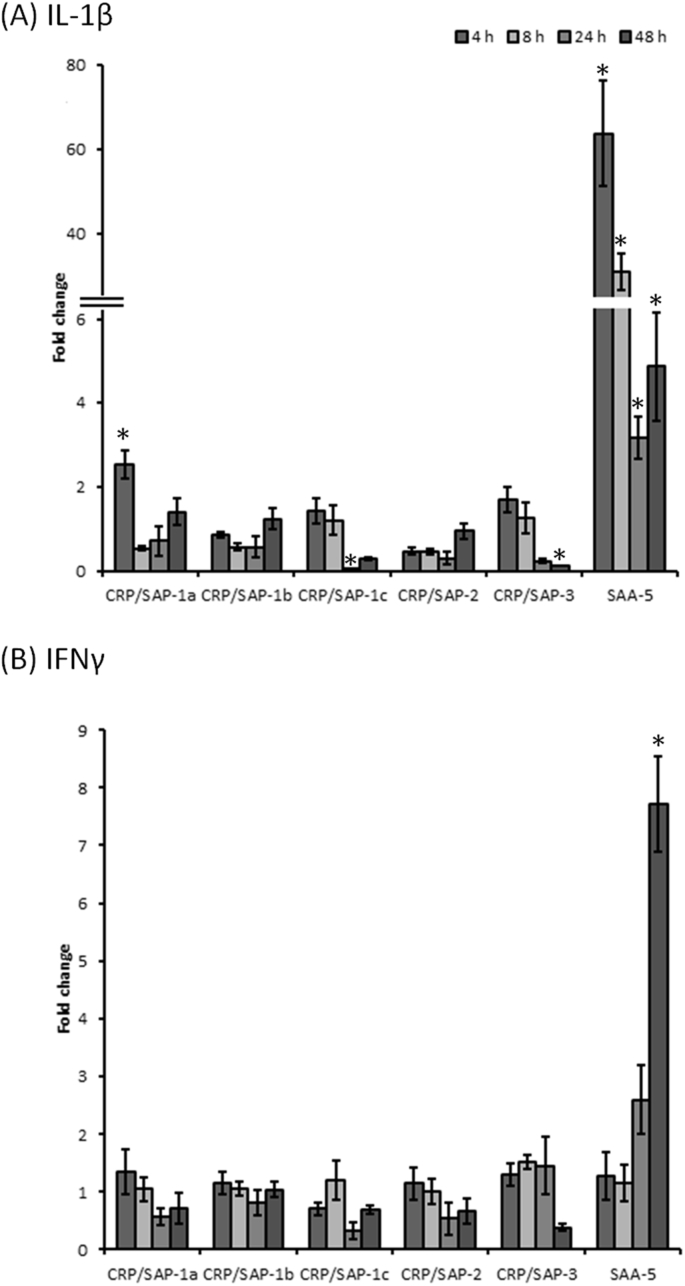
Gene expression of CRP/SAP homologues and *SAA-5* in primary head kidney leukocytes. The cells were treated with medium (control) or medium plus (A) IL-1β (20 ng/ml) or (B) IFNγ (20 ng/ml). The expression levels of CRP/SAP homologues and SAA-5 were normalised to the expression levels of three housekeeping genes (*EF-1α*, *β-actin* and *Hprt1*). The normalised expression levels of the cytokine-treated groups were compared with that of the control groups (n = 3). Statistical analysis was performed using *t*-test. Significant differences (P < 0.05) are indicated by asterisks.

**Fig. 7:**
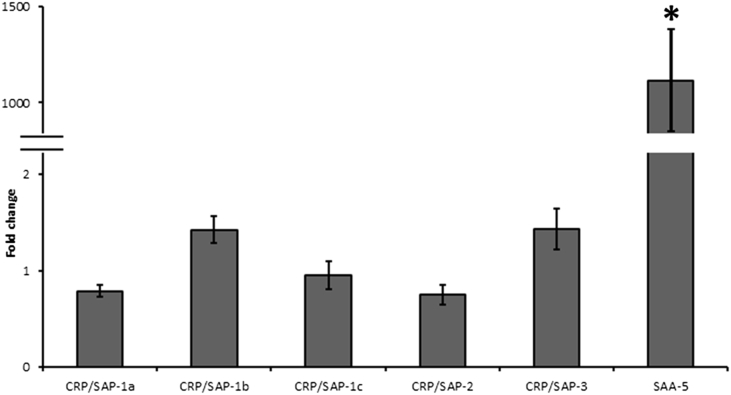
Gene expression of salmon CRP/SAP homologues and *SAA-5* in liver tissues of Atlantics salmon challenged with attenuated *A. salmonicida* (aro A-) strain. The expression levels were normalised to three housekeeping genes (*EF-1α*, *β-actin* and *Hprt1*) and expressed as fold change relative to PBS injected control groups. The expression levels are shown as means ± SE. The *t*-test was used to compare gene expression between the challenged groups (n = 8) and control groups (n = 8). Significant differences (P < 0.05) are indicated by asterisks.

The expression of salmon CRP/SAPs were examined in primary head kidney leukocytes treated with rIL-1β or rIFNγ for 4 h (Fig. 6). In the cells treated with rIL-1β, the salmon *CRP/SAP-1a* was up-regulated. Down-regulation was seen for salmon *CRP/SAP-1c* and *CRP/SAP-3* (P < 0.05) at 8 h, 24 h and 48 h post stimulation (Fig. 6A). In contrast, salmon *SAA-5* transcripts were remarkably increased by rIL-1β at all time points. Treatment of rIFNγ only induced *SAA-5* expression at 48 h whilst did not alter expression of other genes (Fig. 6B). In Atlantic salmon infected with *A. salmonicida* by peritoneal injection, massive induction of salmon *SAA-5* was detected in liver after 24 h but the transcript levels of other *CRP/SAP* homologues remained unchanged (Fig. 7).

## 4 Discussion

The pentraxin-like proteins are evolutionary conserved and are found from the invertebrates to mammals [26]. Indeed, a number of studies have reported the presence of CRP- and SAP-like proteins in teleost fish which were named based on the binding properties compared with their mammalian homologues [3], [11], [33]. In the present study, we report the identification of five pentraxin-like molecules in Atlantic salmon and expression analyses following immune challenges.

Based on the conserved gene structure, it has been suggested that the mammalian *CRP* and *SAP* may have arisen from an ancestral gene duplication event (about 200 million years ago) [37]. Our studies demonstrate that the vertebrate *CRP/SAP* genes comprise either two coding exons or three coding exons, which is in contrast to the horseshoe crab *CRP/SAP* gene which lacks introns [51]. The genomic locations of the salmon genes were as follows; both *CRP/SAP-1a* and *CRP/SAP-2* were located on Chromosome 1, *CRP/SAP-1b* and *CRP/SAP* were on chromosome 19 and the *-CRP/SAP-1c* was on chromosome 2 (Fig. 4). In contrast, the zebrafish *CRP/SAP* genes are located in two chromosomes (Chr. 13 and Chr. 24), with conserved gene synteny between salmon and zebrafish. A *CRP/SAP* gene locus homologous to the one of the two *CRP/SAP* loci in salmon (Chr. 19) and zebrafish (Chr. 24) could also be identified in the spotted gar (LG13) (Fig. 4), providing strong support that they have originated from a common ancestor. The Atlantic salmon has an additional *CRP/SAP* locus (Chr. 02). This may be the outcome of the salmonid specific whole genome duplication, an evolutionary event resulting in extensive expansion of birth of gene paralogs in salmonids [24].

Teleost *CRP/SAP* genes can be divided into two sub-families based on the phylogenetic analysis. The first sub-family (group I) comprises *CRP/SAP* genes from mammals, birds, reptiles, amphibians (except for frog *CRP3*), and piscine species, whilst the second sub-family (group II) consists of the rest of teleost *CRP/SAP* genes examined (mainly from Cyprinid fish), the elephant shark CRPs, the western clawed frog *CRP3* and the coelacanth *CRP* gene. These results suggest that the two *CRP/SAP* families may have diverged before the separation of Holostei and Teleostei. Local random duplications of *CRP/SAP* genes are apparent in some fish species, for example, 7 copies of *CRP/SAP* genes are found to be clustered in zebrafish Chr. 24 (Fig. 4). The phylogenetic analyses also suggest that the avian and mammalian *CRP* and *SAP* genes may have separated more recently from a common ancestral gene (Fig. 3). It has been hypothesised that the mammalian *CRP* and *SAP* genes may have arisen from a gene duplication event which took place about 200 million years ago [37].

In agreement with the observations in humans [47] and goldfish [19], salmon *SAA-5* is found to be widely distributed in tissues examined and appears to be the dominant APP. It is highly expressed in gills, spleen and muscle. Moreover, intraperitoneal injection of the attenuated *A. salmonicida* (aroA(-) strain) induced expression of salmon *SAA-5* in liver, suggesting its important role in APR in Atlantic salmon. Although constitutively expressed in most tissues, other *CRP/SAPs* did not respond to bacterial infection at the transcript level. Previous studies have shown that the expression of *Salmo salar* agarose binding protein (SsABP, identical to *CRP/SAP-1a* in the present study) was also not modulated in fish infected with *A. salmonicida* (strain AL2020) or when injected with formaldehyde inactivated *A. salmonicida* [27]. Likewise, intramuscular administration by injection of *A. salmonicida* (strain 27A) induced *SAA* expression but had no effect on the pentraxin (*CRP/SAP*) in liver tissue of Arctic char (*Salvelinus alpinus*) [15]. Similarly, Ref. [18] detected an increase of the serum CRP level in rainbow trout after exposure to anti-ectoparasitic chemicals such as metriphonate and formalin [18]. However, in common carp, the serum CRP-like proteins were increased after infection with *Aeromonas hydrophila* but not with the *Escherichia coli* (serotype 0111:B4) lipopolysaccharide (LPS) [28]. These observations indicate that the main APR protein in salmon is the *SAA-5* gene, whereas the other genes isolated do not appear to play a role during immune response (at least by the stimulations examined here). There are significant differences in tissue expression which could suggest non-immune but tissue specific functions for these genes. Further research could include promoter analysis of the genes to give indications of the regulatory factors that are involved in driving the gene specific expression and from that infer functional roles. In summary, vertebrate *CRP/SAP* genes have two sub-families, with the salmon *CRP/SAP-1a*, *CRP/SAP-1b*, *CRP/SAP-1c* and *CRP/SAP-2* formed the first *CRP/SAP* subfamily with sequences from different lineage of animals (mammals, birds, reptiles, amphibians and several fish species), and the salmon *CRP/SAP-3* forming the second subfamily with *CRP/SAP* molecules from primary fishes (e.g. Cyprinid fish), spotted gar (*CRP*), frog (*CRP3*) and elephant shark. The expression levels of salmon *CRP/SAPs* were not sensitive to cytokines (rIL-1β and rIFNγ) treatment in primary head kidney cells except that *CRP/SAP-1a* was induced at early time point, and the salmon *SAA-5* was significantly induced by the aforementioned stimulation. Furthermore, salmon *SAA-5* was sharply induced in liver tissues from *A. salmonicida* (aroA(-) strain) challenged fish but the salmon *CRP/SAPs* remained refractory. Hence, more investigations are required to examine the functions of *CRP/SAPs* in fish APR.

## Appendix A Supplementary data

figs1 figs1
