# Supplementary material for: Phylogeny and expression analysis of C-reactive protein (CRP) and serum amyloid-P (SAP) like genes reveal two distinct groups in fish
Source: Fish Shellfish Immunol. 2017 Jun;65:42–51. doi: 10.1016/j.fsi.2017.03.037 (PMC5446266; doi:10.1016/j.fsi.2017.03.037)
Supplement: figs1 [file mmc1.docx]

(A)

>SAA-5

atgaagctgcttctagctggacttgttctgaccctcgttgtaggagctcaagctcagtggtaccgcttccctggtgaagctgctcgag|gtgctaaagacatgtggcgtgcatatggcgacatgaaggacgccaactggaaaaactcagacaagtacttccacgctcggggcaactatgatgctgccaggagaggaccagggggcaggtgggcagcagcagtcatcag|taatggccgggagatggttcagggttccagtggtcgaggacatgaggactcagcagctgaccaggaggctaaccgctggggacgtaatggaggggaccccaaccgctacagaccaaatggactcccaaggaactactga

(B)

CRP/SAP-1a atggagag|tgcactcaatctaatggcgaagctggtgtttttgctgcccctgatctatggc

CRP/SAP-1b atggaaag|tgtactaaagtggatggagaagctgctgtttctgctggttctgacctatggt

CRP/SAP-1c -------- ----------------------------------------------------

CRP/SAP-2 -------- -------------atggagagactctttcttctgccttttctgtttacaatg

CRP/SAP-3 atga---- -----------taactgccaggctcatactcctactgggtatcgtgggcatc

CRP/SAP-1a tgttatggtgaacatcaag|atctctcaggtaaagtgttcgtaatcccaatggcgacaagc

CRP/SAP-1b tgttatggtgaacctcaag|atctctcagggaaaaagttcatcatcccagtcgagacaagc

CRP/SAP-1c ------------------- --atgtcagggaaggaaatcatttccccagtggagtcaaac

CRP/SAP-2 tgctgggctgtgcctcaag|acctctcaggtaaaatgttcacatttccaaaggagtccgat

CRP/SAP-3 acctcagccaataaagttg gcctgggtggaaaggtgctggtcttcccgtatgagacggac

* ** **.. . * .. ..**. * * * ...

CRP/SAP-1a acctcacatgtaaagctccatgcgaacgtctcagagcccatttctgctatgaccatgtgt

CRP/SAP-1b gactcgtttgtaaaactctctgacaacgtcttaaagcctgttattgctatgaccatgtgt

CRP/SAP-1c accgcctatgtcaagataacccctgacatgaacaaaatcttctttgctgtgactatctgc

CRP/SAP-2 tctgatcatgtggtgctaatgcc---aacaggagaaaactattcttctgtgacagtatgt

CRP/SAP-3 ttcagcttcgtggcgctcatcccccaaaaggagatgggcctgcgggcgttcacgctgtgc

. . .** . . * . . . . * * ** * **.

CRP/SAP-1a cagaggttcaactctgagcaaga---acgaggccagtcccttttttctctggcaacccag

CRP/SAP-1b cagaggttcttcactgaggtaca---acgagaccagtcccttttctctctagcaaccccc

CRP/SAP-1c gttagatttttcaccgactaccagacgaaggagctgaccattttctcattggccacgcct

CRP/SAP-2 ctcaggtatttcacagatgtcaa---gagggcgttttccatcttttccatggcaactccg

CRP/SAP-3 atgcgcgtggccaccgagctggagggcgagcggcagatcatcctgttcgcctaccgcacg

* * * ** * .. . .* *..* *. .

CRP/SAP-1a tctcatgacaatgatttgttgttgtacaaacgctccatgggtgtgtaccgagtgcatatc

CRP/SAP-1b tctgattccaaagatatcaatctgtgtctgcaatcaaagggtgggtataaactgaatatc

CRP/SAP-1c tctcatgctaatggttttgttatcttcaggggagatgggggaaattactgggtgtacatt

CRP/SAP-2 acaagcaccaatgacttcctgttgttcaaagagtctaatggtgacatggagctacatgtt

CRP/SAP-3 cccgactacgacgagcttaacgtgtggcgcgagaaagacggccgcattgccttctacctg

* .. ..* *. * * * . . ** * *. *

CRP/SAP-1a aagggagcgtcactggatttcatcagtttgccagattcaaaaaatgaatggatctccatc

CRP/SAP-1b agaggaaattctgttactatcaacggtttgccagaaaatagaaatggatggatctctttt

CRP/SAP-1c ggggaccaaggtatctatttctggggattgccagacaaaatgaacgagtggaactctgtt

CRP/SAP-2 agatatgtgggaagtgcattcacagggctaccaggtgagcagaacatgtggatgtctctg

CRP/SAP-3 agtggcgacggcgccttcttccacctgccgcccatcaccacattca---gaacaagtctc

.. . ** ...** . . .. *.* . *

CRP/SAP-1a tgctggacctgggactctaaaagtggtctgacccagctgtgggttaatgggaagcgaagt

CRP/SAP-1b tgtgtcacgtgggactctaaaactggtctcacccagatgtgggcaaatgggagacgaagt

CRP/SAP-1c tgtgggacgtgggatgccagtacaggattgactcaactgtgggtgaatgggaagccaagt

CRP/SAP-2 tgtgggagctgggactcagtcaccggactcagtcaggtttggatcaatgggaagccgagt

CRP/SAP-3 tgcctcacctgggagtcccttaccgggctggctgccttttgggtggacggccgccgcagc

**. * ***** * * ** .* . . * ***.. .*.** . * **.

CRP/SAP-1a gcacggaggattcttaaacctgatacatctgta---actggcacaccaagtataatgtta

CRP/SAP-1b gcatcgaagattcttaagccaaatggccctata---aatggaaaaccaagtataatctta

CRP/SAP-1c gcgaggaaagctctccaagccggcggctccatc---tctggtactctgagtattatttta

CRP/SAP-2 gcaaggaagatgggatacactgctgggagtgtgctgaatggaaaacccatcatcatccta

CRP/SAP-3 acctaccaggtgtacaagccgggccacaccatc---cggcccaaggggaccgtcctcctc

.* .... * * . . ..* * * ..* * .*

CRP/SAP-1a gttcaagagcaagacagttatggcggaggttttgatgtctcacaatcctttgtgggggag

CRP/SAP-1b ggtcaaaaccaaggcagttatggtggaggttttgtagcatcacaatcctttgtgggggat

CRP/SAP-1c ggtcaggcccaagatgcatatgctggtgggtttgatgtgaatgactccttttacggacat

CRP/SAP-2 ggccaggaccaggattcttatggtggaaatttcgataagggacaagcgtttattggtcaa

CRP/SAP-3 gggcaagacccggacaaacacctgggagacctggaggcagtgcagagctttgtgggggaa

* **.. * .*.. .*. ** .. .* * . * *** ** *

CRP/SAP-1a gttactgacgtccacttctgggacagtgtcatctctccctgtgaaatacaattgtatatg

CRP/SAP-1b gttactgatgtacacttctgggacagtgtcatctctccttgccaaatcaaattgtatatg

CRP/SAP-1c gagaccgatgtccacatgtgggacagagtgctctccccatgtgagatccaaagctacatg

CRP/SAP-2 ctcactgatgtgcacatgtggaattatggtctgtcaccttgtgagatccagcgcttcaca

CRP/SAP-3 gtgactgacctcaacatgtgggactatgtgctgcccagaagccagatccaagccctgcat

**.**. * ** * ***.*. . * * .* *. *.** *. .

CRP/SAP-1a cagttgaatagatttactccaggaaatattctcaactggaaagcattggagttcactgtt

CRP/SAP-1b caagggaaaaactttactccaggaaatattctcaactggaaagcattggagttcactact

CRP/SAP-1c aagggggaggtgttatctccagggaatgtggtaaactggaacgcactaaaatacacaagg

CRP/SAP-2 agcgacttaaatttcacccctgggaatgtacttaactggaggtcccttgagtacacaatt

CRP/SAP-3 tacgaacataagatctccaaaggaaacatctttgactgggctaccatcgagtaccagctg

. . * *. **.**..* * .*****. * * .*.* *

CRP/SAP-1a gagggaaaagtgttcatagagagaa---gtgaatttagaagtgaatgttataattattag

CRP/SAP-1b gggggaggggtgttcaaggagataa---gtgactatacctgttag---------------

CRP/SAP-1c catggctatgtggttgttgagaggatgcagaacctgatctgttaa---------------

CRP/SAP-2 gatgggcatgtggttctcgaaaacaagcaaacaccttgccaagaa------------tga

CRP/SAP-3 aacgggaatgtgatggtggtggatg---atgactga------------------------

. ** . *** * * .. . . . .

Supplementary Fig. 1. Primers location of salmon *SAA-5* (A) and *CRP/SAPs* (B). The intron-exon junctions were labeled as “|” and the primer sequences were underlined. The cDNAs of salmon CRP/SAPs were aligned using the ClustalW programme. Alignment gaps are inserted by dashes in the nucleotide sequences. Identical residues are represented by an asterisk (*), conservative substitutions by a single dot (.), and highly conservative substitutions by colons (:).
